# Changing the paradigm of intracranial hypertension in brain tumor patients: a study based on non-invasive ICP measurements

**DOI:** 10.1186/s12883-020-01837-7

**Published:** 2020-07-06

**Authors:** Jenny C. Kienzler, Rolandas Zakelis, Serge Marbacher, Sabrina Bäbler, Lucia Schwyzer, Elke Remonda, Javier Fandino

**Affiliations:** 1 Department of Neurosurgery, Kantonsspital Aarau, Tellstrasse, CH-5001 Aarau, Switzerland; 2 Kaunas University of Technology, Health Telematics Science Institute, Kaunas, Lithuania

**Keywords:** Brain tumor, Intracranial hypertension, Intracranial pressure, Non-invasive ICP measurement

## Abstract

**Background:**

The ultrasound based non-invasive ICP measurement method has been recently validated. Correlation of symptoms and signs of intracranial hypertension with actual ICP measurements in patients with large intracranial tumors is controversial. The purpose of this study was to assess ICP in patients with brain tumors, presenting with neurological signs and symptoms of elevated ICP and to further evaluate the value and utility of non-invasive ICP monitoring.

**Methods:**

Twenty patients underwent non-invasive ICP measurement using a two-depth transcranial Doppler ultrasound designed to simultaneously compare pulse dynamics in the proximal (intracranial), and the distal (extracranial) intraorbital segments of the ophthalmic artery through the closed eyelid.

**Results:**

Forty-eight measurements were analyzed. Radiological characteristics included tumor volume (range = 5.45–220.27cm^3^, mean = 48.81 cm^3^), perilesional edema (range = 0–238.27cm^3^, mean = 74.40 cm^3^), and midline shift (mean = 3.99 mm). All ICP measurements were in the normal range of 7–16 mmHg (ICP_mean_: 9.19 mmHg). The correlation of demographics, clinical and radiological variables in a bivariate association, showed a statistically significant correlation with neurological deficits and ICP_max_ (*p* = 0.02) as well as ICP_mean_ (*p* = 0.01). The correlation between ICP and neurological deficits, showed a negative value of the estimate. The ICP was not increased in all cases, whether ipsilateral nor contralateral to the tumor. The multivariate model analysis demonstrated that neurological deficits were associated with lower ICP_max_ values, whereas maximum tumor diameter was associated with larger ICP_max_ values.

**Conclusions:**

This study demonstrated that ICP in patients with intracranial tumors and mass effect is not necessarily increased. Therefore, clinical signs of intracranial hypertension do not necessarily reflect increased ICP.

## Background

For decades, neurosurgeons and neuro-oncologists assumed that the mass effect of brain tumors with peritumoral edema or intratumoral hemorrhage leads to increased intracranial pressure (ICP) [1]. This assumption has been fundamental to the management not only of brain tumors but also, by extrapolation, of intracranial mass lesions in general. Critical management decisions including the timing and nature of surgical procedures and medical intervention have come to be based upon and driven by clinical and radiological findings associated with increased ICP.

Quite surprisingly, there is remarkably little evidence supporting the assumption that intracranial lesions with mass effect categorically result in raised ICP. On one hand, raised ICP is certainly associated with a number of historically pathognomic signs and symptoms including headache, nausea, vomiting, papilledema and neurological deficits [2–5]. On the other hand, there is a dearth of quantitative correlation. This assumption is more difficult to confirm than one might think.

A medical device allowing non-invasive ICP measurement without a relevant risk of side effects could substantially add to our knowledge of ICP dynamics in patients in whom invasive measurement is not otherwise clinically warranted. This group includes many patients with newly diagnosed brain tumors presenting with clinical and radiological signs of intracranial hypertension. Having this in mind, our group recently published the results of a validation pilot study on non-invasive ICP measurement method including 78 simultaneous paired invasive and non-invasive ICP values [6]. In this study, no significant difference between the two groups could be found and the accuracy of this technique in this pilot study was − 1.130 mmHg [6].

The overall aim of the current study was to a) explore the relationship between intracranial tumor volume and ICP as well as b) identify the clinical factors associated with increased ICP.

## Methods

### Patient selection

All patients with intracranial tumors, and signs of mass effective brain tumors were screened for study inclusion between December 2014 and January 2016. Mass effect was defined in terms of clinical (headache, nausea, vomitus, neurological deficits) and/or radiological (perilesional edema, midline shift, and concomitant hydrocephalus) signs of intracranial hypertension.

The study was approved by the local ethics commission (EKNZ Nr.2016–00507). Patients were either referred to our emergency department or admitted for elective surgery. All patients or their family provided written voluntary consent prior to the study enrollment.

### Technical background

The ICP measurement instrument used in this trial (Vittamed™ 205, Vittamed Boston Neurosciences Corporation, Lexington, Massachusetts) was developed at the Health Telematics Science Institute at the Kaunas University of Technology, Lithuania [7]. A pilot study for validation of this non-invasive ICP measurement technique has been published recently with a detailed method description [8].

The measurement technique is based on the use of transorbital Doppler ultrasound of the ophthalmic artery (OA), as a natural ICP sensor. The OA has two major segments. The first, proximal or intracranial segment originates at the carotid artery and extends to the optic canal (OC), at which point it perforates the dura. The second, distal or intraorbital segment originates distal to the OC and accompanies the optic nerve within the orbit to the retina.

Although the proximal and distal OA are adjacent and continuous, they have different pulse dynamics. These differences, which can be demonstrated in waveform measurements obtained using transorbital Doppler ultrasound, reflect differences in transmural pressure. Inasmuch as there are no other pertinent physiological or anatomical factors distinguishing the proximal and distal segments of the OA or contributing to the differences in transmural pressure, the differences in pulse dynamics are attributed to the influence of ICP. Proximal to the canal, the OA is subject to ICP. Distal to the canal, in the orbit, it is not.

These characteristics serve as the basis for non-invasive ICP monitoring. Insofar as the tissues of the orbit are non-compressible, pressure applied to the orbit is transferred to the distal OA. It is possible to balance the pulse dynamics of the proximal and distal OA through the application of gentle pressure to the orbit. When the pressure applied equals the ICP, the pulse dynamics and the waveforms equilibrate. The pressure required to reach the point of equilibration, the “balance point,” is equal to the ICP and can be read out from the ICP monitoring instrument.

Inasmuch as neither individual patient calibration nor zero-level calibration is required, this method can be said to deliver a measurement of absolute, rather than relative intracranial pressure.

**Fig. 1:**
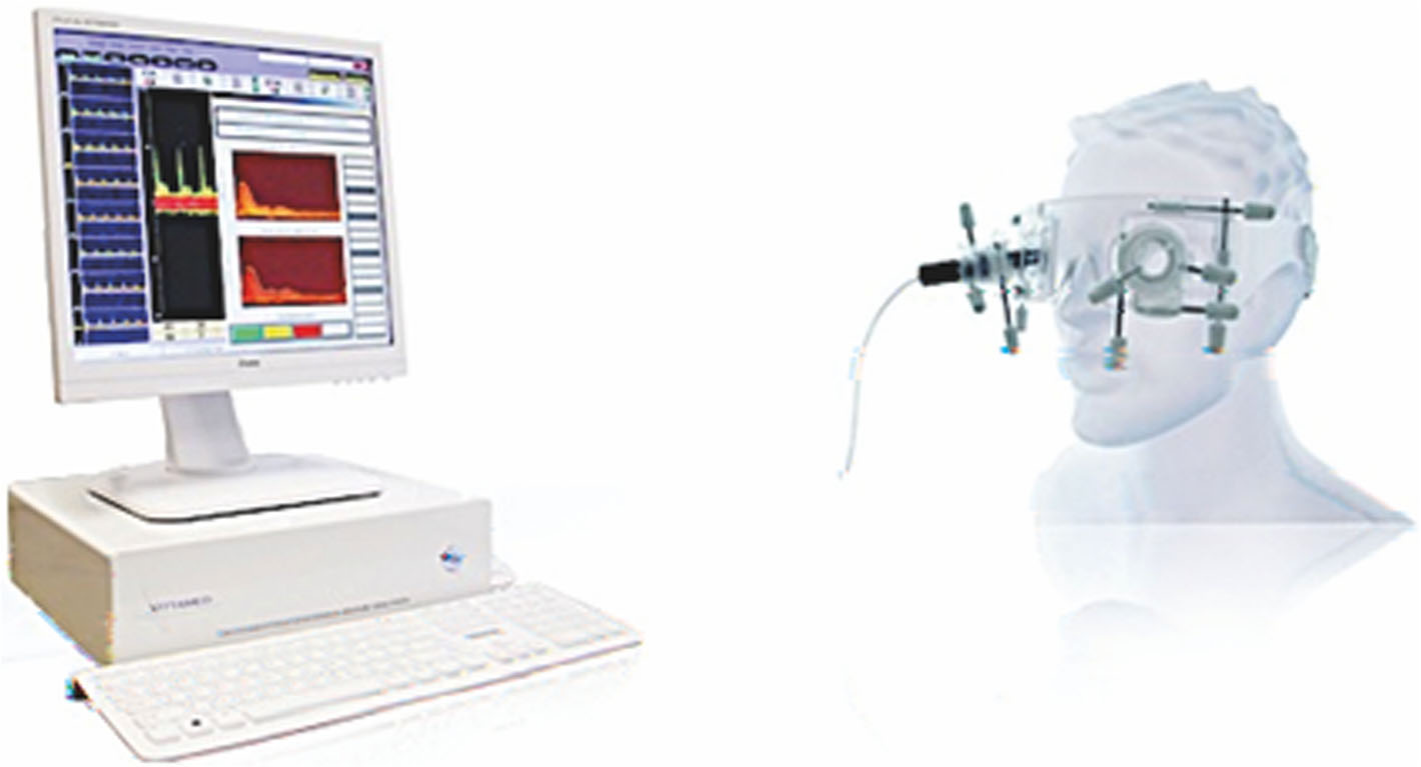
The non-invasive ICP measurement device (Vittamed™ 205). Source and copy right: Vittamed. Permission to use and adapt obtained from: Prof. Arminas Ragauskas, DSc, FBC, FLSHD, Head of Health Telematics Science Institute at Kaunas University of Technology, Kaunas, Lithuania

In this study, pulse dynamics were assessed using a customized trans-orbital Doppler ultrasound device and a narrow tubular two-depth single beam transducer. The transducer is inserted into a fitting on a plastic frame strapped around the head (Fig. 1). The fitting is engineered to allow considerable adjustment and freedom of movement. The ultrasonic transducer is first used to scan the orbit in order to confirm the position of both segments of the OA, and then aimed and adjusted to optimize signal strength and quality. The width of the ultrasound beam is sufficient to insonate both segments of the OA simultaneously.

Measurements may be taken from either side. The side chosen most often reflects the preferences of the examiner unless previous orbital injury or other ocular history presents a contraindication to one side. Otherwise the second side is reserved for re-measurement and confirmation, or in the event of failure to complete the measurement.

An air-inflatable doughnut shaped cushion is positioned between the frame and the orbit on the side chosen for measurement. The cushion is used to apply pressure to the orbit. The transducer passes through the opening in the cushion, positioned against the closed eyelid, aimed and then fixed in place. Pressure in the cushion is measured continuously in mmHg throughout the measurement procedure. The ICP measurement procedure is fully automated from the point at which the position of the ultrasonic transducer has been optimized and fixed.

The actual measurement technique involves the following steps: pressure is applied to the tissues surrounding the eyeball, and increased automatically in steps of 2.0 or 4.0 mmHg. The maximal duration of each step is limited to 40 s. The software built into the ICP meter automatically detects the point of equilibrium at which the waveforms of the proximal and the distal segments of the OA are matched. The amount of pressure externally applied to the orbit at this point equals the ICP.

### Measurements

We attempted to perform non-invasive ICP measurements in all patients on both eyes and if patient compliance allowed, multiple measurements were done. All measurements were obtained in supine position by experienced neurovascular doppler ultrasound technicians with specific training for the non-invasive ICP device.

Out of 53 measurement attempts, 48 measurements were reliable and repeatable and were therefore included in this analysis (Supplementary Table 1). In four cases, the measurement could be completed in only one eye. A total of 5 measurements had to be excluded due to device or patient related causes: 1) because of a device related technical limitation (unable to identify a reliable ICP balance point) 2 + 3) during these measurements, poor or unstable TCD signal impeded a final result (patients eye movement), 4) due to interfering extrasystolic beats during measurement no reliable result could be achieved, 5) another patient suffered uncontrollable hiccups and therefore could not hold still, which is mandatory during measurement.

### Radiographic and clinical assessment

All patients underwent detailed case-history and neurological examination prior to the measurement with special attention to the classic signs of raised intracranial pressure including headache, vomiting or impaired consciousness. All patients underwent magnetic resonance imaging (MRI) for preoperative morphologic evaluation and intraoperative navigation purposes. Evaluation of the MRI included signs of intracranial hypertension: Tumor size and volume, peritumoral brain edema volume, and midline shift (MLS). Midline shift was measured at the level of the foramen of Monro. Volumetric measurements of the tumor mass and perifocal edema (cm^3^) were performed using Elements software (BrainLab®, Munich, Germany). The investigators performing the imaging analysis were blinded to the clinical findings and non-invasive ICP values.

### Statistical analysis

The primary endpoint was set as the maximum ICP value (ICP_max_) for each patient. Secondary endpoints included ICP on the affected side of the brain (ICP_aff_): Mean ICP in left hemisphere for left-sided tumors and on the right for right sided tumors. At the same time arithmetic mean overall ICP value (ICP_mean_) for each patient as well as individual ICP values for each side of each patient were calculated.

We used a bivariate association analysis to investigate associations between maximum ICP value (ICP_max_) and all other variables. Associations of ICP_max_ with categorical explanatory variables were investigated by Wilcoxon rank sum tests (Mann-Whitney test), estimating the difference between groups together with a 95% confidence interval. Associations of the ICP_max_ with continuous explanatory variables were investigated with linear regression, estimating the effect size together with a 95% confidence interval. In order to achieve an optimal multivariate model to explain the ICP_max_, the ICP_aff_ and the ICP_mean_, a model with a maximum of two explanatory variables was evaluated and a forward-selection approach was used based on the Akaike information criterion to find the most relevant explanatory variables.

## Results

### Patient demographics

**Fig. 2:**
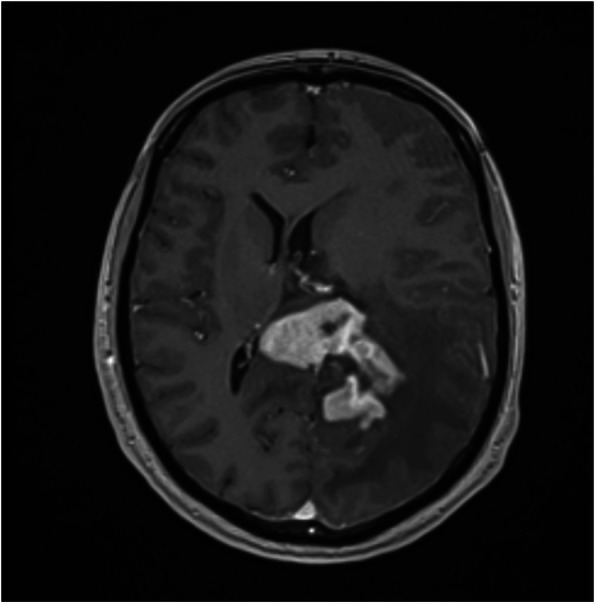
Illustrative Case. Preoperative MRI of patient Nr. 18, showing a parietooccipital left sided intra-axial lesion with infiltration of the splenium of the corpus callosum. The lesion is centrally necrotic with dense contrast enhancement around the margins. The measured tumor volume is 36.5 mm^3^ and edema volume 231.4mm^3^ with 15 mm midline shift and consecutive hydrocephalus. Clinically the patient initially presented with a Gerstmann syndrome and neurocognitive deficits. The preoperative ICP was measured 9.08 mmHg on the left side and 11 mmHg on the right side. A performed biopsy diagnosed a B-cell type non-hodgkin lymphoma

A total of 20 patients (12 male) were included in the final analysis of this pilot study. The mean age at the time of surgery was 63.8 ± 13.2 years (range 41–83 years). The majority of tumors were located supratentorial and only three were infratentorial. Most common histological tumor diagnosis was meningioma followed by glioma, and metastases. Demographic and patients’ characteristics are shown in Table 1. Five patients underwent a biopsy procedure only. An illustrative case is shown in Fig. 2. This patient was referred to our department due to a newly diagnosed cerebral lesion and clinical history of slowdown, aggressive behavior and depression. Clinical examination revealed a Gerstmann syndrome with an initial GCS of 15. While the patient was waiting for the biopsy surgery, a sudden deterioration with a new right sided hemiparesis, vomitus and impaired consciousness occurred. There was no evidence for the presence of epileptic seizures. The measured ICP was 11 mmHg. After admission to ICU, application of hypertonic saline and mannitol with head increased position, patient regained a GCS of 15 and biopsy obtained a lymphoma.

**Table 1:** Patient demographics, clinical and radiological findings

Patient Number	GCS	Neurological deficits	Steroids	Clinical signs of ICH	Tumor location	Histology	Concomitand disease	Maximal tumor diameter (cm)	Tumor structure	Tumor volume (cm^**3**^)	Edema volume (cm^**3**^)	Midline shift	Hemorrhage	Hydrocephalus	Non-invasive absolute ICP left / right
**1**	15	apraxia, visual field restriction left side, vision impairement left side	no	none	Bilateral parafalcine occipital	Meningeoma Grade II	Hypertension Diabetes mellitus Typ II	4.7 × 5.4 × 8.1	solid with homogeneously contrast enhancement	106.226	151.439	2.5 mm	none	none	10.4 / 6
**2**	15	absences, focal seizures	no	none	Left frontal	Diffuse Astrocytoma Grade II	none	2.6 × 4.0 × 5.0	T2 hyperdens / T1 hypodens lesion without contrast enhancement	33.723	0.000	none	none	none	9.94 / 7.95
**3**	14	focal and secondarily generalized seizures	yes	none	Left temporal	Glioblastoma multiforme	Hypertension Alcohol abuse	4.0 × 2.7 × 3.9	T2 hyperdens / T1 hypodens lesion without contrast enhancement	24.985	0.000	none	none	none	7.14 / 10.43
**4**	15	focal seizures	no	none	Multifocal (right thalamus, left parietal, right frontal right)	Astrocytoma Grade III - IV	Metabolic syndrome GIST Tumor of the stomach	3.1 × 3.1 × 4.0	multifocal central necrotic lesion with margin contrast enhancement	30.217	89.075	3 mm	none	none	10.09 / 9.15
**5**	15	absences, memory disorders	yes	none	Left temporal	Meningeoma Grade I	none	2.5 × 3.1 × 2.5	solid with homogeneously contrast enhancement	10.306	115.310	5.3 mm	none	none	10.82 / -
**6**	15	gerstmann syndrome, aphasie	no	none	Left temporo-occipital	Metastasis	Renal cell carcinoma	1.9 × 2.0 × 2.2	central necrotic lesion with homogeneously contrast enhancement	5.450	67.337	none	yes	none	7.0 / -
**7**	15	vertigo	yes	headache, nausea, vomitting	Right cerebellum	Metastasis	Hypertension Breast cancer	3.7 × 4.2 × 2.7	solid with moderate non homogeneously contrast enhancement	23.783	10.304	none	none	none	10.5 / 12.71
**8**	14	neglect, apraxia	no	none	Right parietal	Glioblastoma multiforme	Metabolic Syndrom Coronary heart disease Cerebrovascular and peripheral occlusive disease COPD	3.7 × 6.2 × 5.5	central necrotic lesion with margin contrast enhancement	62.100	47.995	2 mm	none	none	12.57 / 11.43
**9**	14	neurocognitive deficits	no	none	Bilateral frontal parafalcine	Meningeoma Grade I	none	5.7 × 4.6 × 5	solid with homogeneously contrast enhancement	56.933	0.000	9 mm	none	none	5.5 / 9.9
**10**	14	neurocognitive deficits	no	none	Left Temporal	Meningeoma Grade I	Metabolic Syndrom Breast Cancer Status following pulmonary embolism	3.9 × 3.5 × 2.8	solid with homogeneously contrast enhancement	23.421	2.325	none	none	none	10.45 / 16.72
**11**	15	none	yes	none	Right frontal	Meningeoma Grade I	Coronary heart disease Status following pulmonary embolism	2.1 × 2.4 × 1.9	solid with homogeneously contrast enhancement	7.077	117.318	none	none	none	5.52 / 5.53
**12**	15	vertigo, unsteadiness, diplopia	yes	headache	Vermis cerebelli	Metastasis	Breast Cancer	4.2 × 3.7 × 3.8	solid with moderate contrast enhancement	25.550	36.790	none	none	yes	5.59 / 10.70
**13**	15	hemiplegia right side	yes	none	Left frontal	Metastasis	Prostate Cancer Squamous skin cancer Metabolic syndrome Malignant melanoma	2.2 × 3.8 × 3.1	central necrotic lesion with margin contrast enhancement	11.619	45.586	none	none	none	7.59 / 5.22
**14**	15	vertigo	yes	headache	Left cerebellum	Meningeoma Grade I	TIA prior to admission	3.5 × 4.2 × 3.9	solid with homogeneously moderate contrast enhancement	26.960	8.591	none	none	yes	11.0 / 6.0
**15**	15	hemiparesis right side, unsteadiness	yes	none	Left parietal	Glioblastoma	Hypertension	3.6 × 5.3 × 4.6	central necrotic lesion with margin contrast enhancement	50.717	85.340	none	none	none	8.0 / 8.1
**16**	14	hemiparesis left side, focal seizures, neurocognitive maladjustment	no	none	Bilateral Corpus Callosum	Non - Hodgkin Lymphoma B-cell type	Delusional disorder	5.2 × 3.5 × 2.3	solid with inhomogeneously contrast enhancement	31.731	196.777	none	none	none	8.34 / 8.16
**17**	14	hemiparesis left side, neurocognitive deficits	yes	headache	Right Parieto-occipito-temporal	Metastasis	Schizophrenia Lung carcinoma	7.6 × 6.0 × 5.7	central necrotic lesion with margin contrast enhancement	177.348	18.703	14 mm	yes	yes	- / 10.67
**18**	15	gerstmann syndrome, neurocognitive deficits	no	none	Left parieto-occipital left with infiltration of Splenium Corpus callosum	Non - Hodgkin Lymphoma B-cell type	Coronary atherosclerosis	5.8 × 5.2 × 3.6	central necrotic lesion with thick margin contrast enhancement	36.495	231.438	15 mm	none	yes	9.08 / 11.00
**19**	15	neurocognitive deficits, incoordination	yes	headache	Right frontal right side with infiltration of the thalamus	Astrocytoma Grade III	None	6.1 × 8.0 × 8.8	central necrotic and cystic wide spreading, diffuse lesion with only punctual contrast enhancement	220.271	25.399	13 mm	none	yes	6.0 / 7.5
**20**	15	none	yes	headache	Right occipito-temporal	Metastasis	Pancoast tumor	2.2 × 2.9 × 3.1	solid with inhomogeneously contrast enhancement	11.299	238.269	16 mm	none	none	14.5 / -

*Abbr.: GCS* Glasgow Coma Scale, *ICH* intracranial hypertension, *ICP* Intracranial Pressure

### Clinical and radiological findings

All patients were awake and cooperative during the non-invasive ICP measurements. Neurological assessment categorized all patients as GCS 14 or 15. Preoperative common symptoms included seizures, neurocognitive and sensorimotor deficits. Potential clinical signs of raised intracranial pressure such as headache were found in 6 patients (30%). In our series, 11 patients (55%) had been placed on steroids a few days prior to non-invasive ICP measurement as a treatment of symptoms as well as preparation for surgery due to pertinent edema in the MRI findings.

Mean tumor volume was 48.81cm^3^ (range 5.45–220.27cm^3^) and mean edema volume was 74.40cm^3^ (range 0 to 238.27cm^3^). A large edema volume of >100cm^3^ was present in 6 (30%) cases. Histologically this subgroup included 3 meningiomas, 2 lymphomas, and one metastasis.

Overall mean midline shift (MLS) was 4 mm. The MLS ranged from an absent MLS to a maximum of 16 mm, which was present in 9 patients (45%). Tumor and edema volumes are shown in Table 2. Additional radiological findings include 2 tumors (10%) with intralesional hemorrhage and 5 (25%) patients with hydrocephalus. Of note, 80% of cases with hydrocephalus were symptomatic with headache.

**Table 2:** Continuous variables of patients in the study cohort

Variable	Mean	SD	Median	Min	Max
Tumor volume cm^3^	48.81	56.77	28.59	5.45	220.27
Edema volume cm^3^	74.40	77.80	46.79	0.00	238.27
Tumor + edema volume	123.21	87.21	114.69	24.98	267.93
Midline shift mm	3.99	5.88	0.00	0.00	16.00
Max tumor diameter cm	4.84	1.80	4.20	2.20	8.80
ICP_aff_	8.81	1.51	8.30	5.53	11.00
ICP_max_	10.65	2.75	10.68	5.53	16.72
ICP_mean_	9.19	2.19	8.81	5.53	14.50

*Abbr.: n* number of patients with measurement, *SD* standard deviation, *min* minimum, *max* maximum

Non-invasive ICP measurements in the current study cohort were in the normal range between 7 and 16 mmHg. Overall, the ICP_mean_ was 9.19 mmHg, the mean ICP_max_ was 10.65 mmHg, and the mean ICP on the affected hemisphere side (ICP_aff_) was 8.81 mmHg (Table 3).

**Table 3:** Bivariate associations of maximum ICP, ICP on the affected side of the brain, mean ICP and individual measurements of ICP (left and right side per patient) with categorical explanatory variables

Variable	%	ICP_**max**_ ES [95% CI]	***P***-value ICP_**max**_	ICP_**aff**_ ES [95% CI]	***P***-value ICP_**aff**_	ICP_**mean**_ ES [95% CI]	***P***-value ICP_**mean**_	ICP_**all**_ ES [95% CI]	***P***-value ICP_**all**_
Sex (male vs. Female)	60	−0.04 [−2.48, 3.47]	0.97	0.07 [−2.05, 2.06]	0.97	0.90 [−1.12, 2.72]	0.31	0.44 [−1.32, 2.20]	0.605
Intraaxial	70	−0.91 [−4.57, 2.06]	0.40	− 0.84 [− 2.47, 1.47]	0.31	− 0.10 [− 2.91, 2.53]	0.97	− 0.30 [− 2.19, 1.59]	0.743
Neurological Deficits	90	−5.81 [−9.13, − 1.79]	**0.02**	− 2.18 [− 4.92, 0.55]	0.32	−5.39 [−8.06, −3.12]	**0.01**	−5.05 [−7.94, − 2.15]	**0.002**
Steroids	55	− 0.93 [− 4.18, 1.10]	0.50	− 0.11 [− 2.17, 1.25]	0.74	−0.78 [− 2.52, 1.05]	0.37	−1.25 [− 2.80, 0.30]	0.107
Clinical signs of ICH	30	1.00 [−1.57, 3.74]	0.31	1.05 [−0.11, 2.97]	0.12	0.45 [−1.48, 2.97]	0.60	0.02 [−1.87, 1.91]	0.983
Hemorrhage	10	−1.95 [−7.50, 2.57]	0.32	−0.13 [−3.47, 3.53]	1.00	−0.20 [−3.83, 3.17]	1.00	− 0.33 [− 4.33, 3.66]	0.864
Hydrocephalus	25	0.27 [−3.50, 3.08]	0.80	0.72 [−1.37, 2.72]	0.23	0.11 [−2.32, 2.10]	0.87	−0.28 [− 2.28, 1.72]	0.771
Age (> 60 y vs. ≤ 60 y)	55	−1.73 [− 1.57, 3.68]	0.20	−0.55 [− 1.37, 2.42]	0.36	0.73 [− 1.20, 2.62]	0.33	0.21 [− 1.53, 1.95]	0.799
Cerebrum vs. Cerebellum	85	−1.08 [−4.37, 3.50]	0.31	−1.05 [−3.35, 1.47]	0.24	0.36 [− 1.77, 2.53]	0.76	0.41 [− 1.83, 2.64]	0.707
Solid vs. Necrotic tumor	60	1.88 [−0.81, 4.00]	0.13	0.24 [−1.64, 1.92]	0.68	0.55 [−1.74, 2.45]	0.68	0.33 [−1.45, 2.11]	0.701
Histology									
Glioma	30	NA [NA, NA]	0.63	NA [NA, NA]	0.56	NA [NA, NA]	0.95	0.41 [−1.86, 2.69]	0.707
Meningioma	30								
Metastasis	40								
Tumor location									
left	45	NA [NA, NA]	0.92	NA [NA, NA]	0.87	NA [NA, NA]	0.85	0.27 [−2.04, 2.57]	0.81
right	30								
bilateral	25								
Tumor volume (cm^3^)		0.00 [−0.02, 0.03]	0.84	0.01 [−0.01, 0.02]	0.31	0.00 [−0.02, 0.02]	0.955	−0.00 [− 0.02, 0.01]	0.864
Edema volume (cm^3^)		0.00 [−0.02, 0.02]	0.84	−0.00 [− 0.01, 0.01]	0.76	0.01 [− 0.01, 0.02]	0.218	0.01 [− 0.01, 0.02]	0.308
Midline shift (mm)		0.09 [−0.13, 0.32]	0.39	0.05 [−0.09, 0.20]	0.43	0.16 [−0.01, 0.32]	0.066	0.10 [−0.07, 0.26]	0.230
Tumor+edema vol. (cm^3^)		0.00 [−0.01, 0.02]	0.75	0.00 [−0.01, 0.01]	0.66	0.01 [−0.01, 0.02]	0.257	0.00 [−0.01, 0.01]	0.447
Max. tumor diam. (cm)		0.33 [−0.41, 1.07]	0.36	0.31 [−0.09, 0.71]	0.12	0.10 [−0.50, 0.70]	0.720	0.11 [−0.39, 0.61]	0.658

*Abbr: CI* confidence intervals, *ES* estimated effect size, *ICP*_*all*_ all individual ICP measurements, *NA* not available

Regarding histological subgroups, mean ICP_max_ in the meningioma cohort was 10.7 mmHg and 9.8 mmHg in glioma patients. The highest measured ICP of 16.72 mmHg was measured in a patient with a grade I meningioma with a tumor and edema volume of 23.4cm^3^, respectively 2.3cm^3^ and no MLS. Patients with radiological signs of hydrocephalus had a mean ICP_max_ of 10.2 mmHg and mean ICP_max_ was 10.5 mmHg in cases without steroid intake prior to measurement.

### Correlation of demographics, clinical and radiological findings with ICP

#### Bivariate associations

**Fig. 3:**
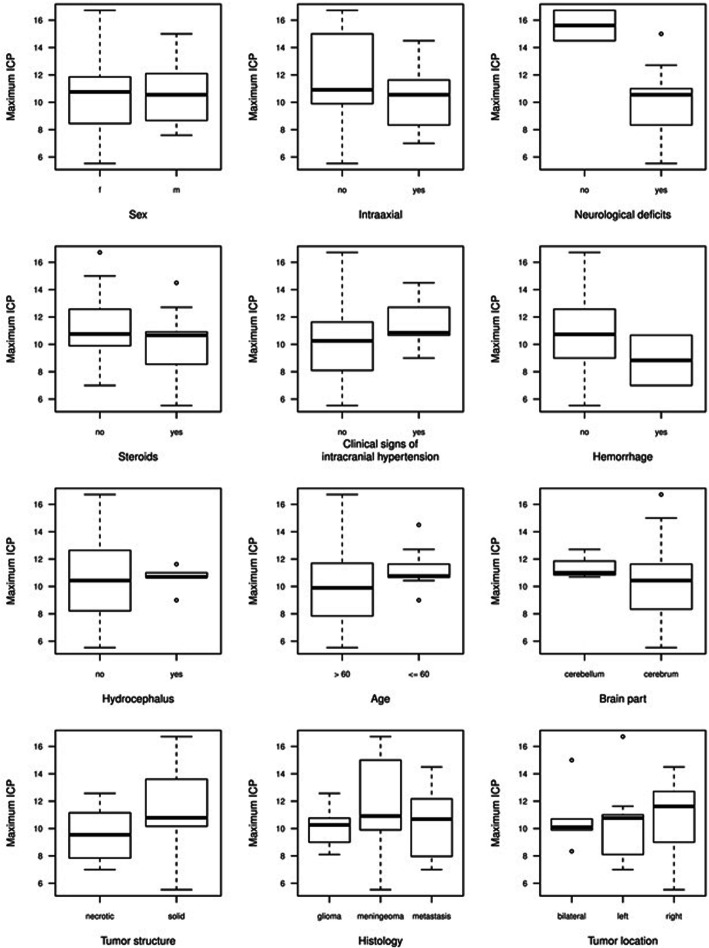
Boxplots of maximum ICP by all categorical explanatory variables. Abbr: f = female, m = male

**Fig. 4:**
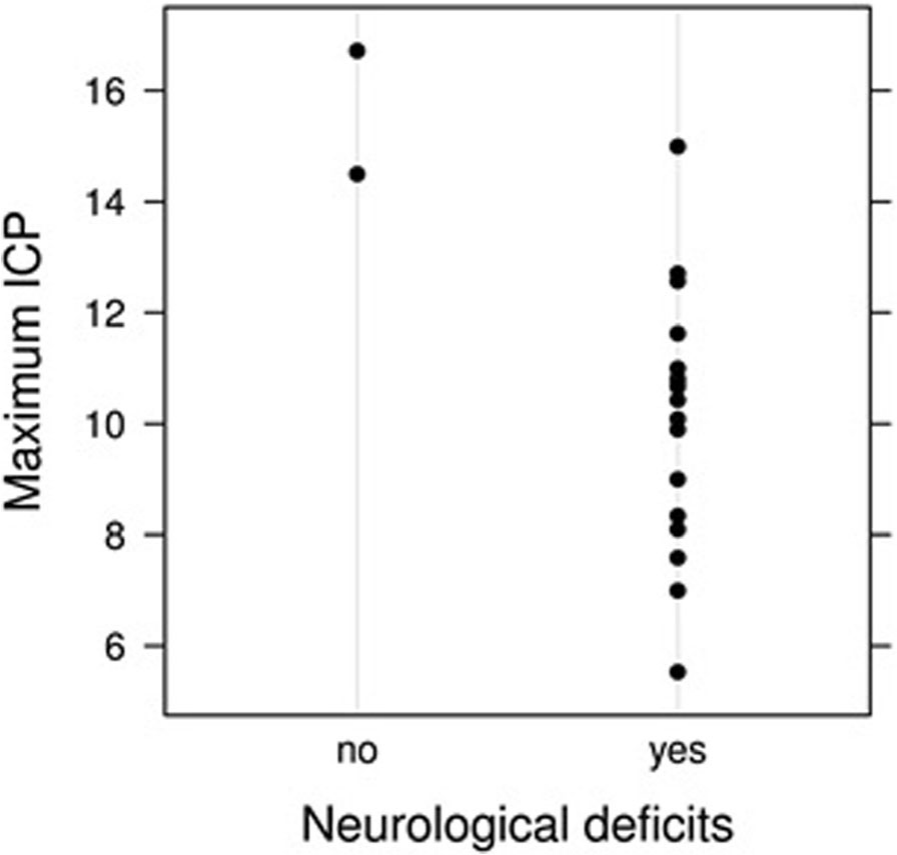
Dotplot of maximum ICP vs. neurological deficits. Note that there were only two patients without neurological deficits

**Fig. 5:**
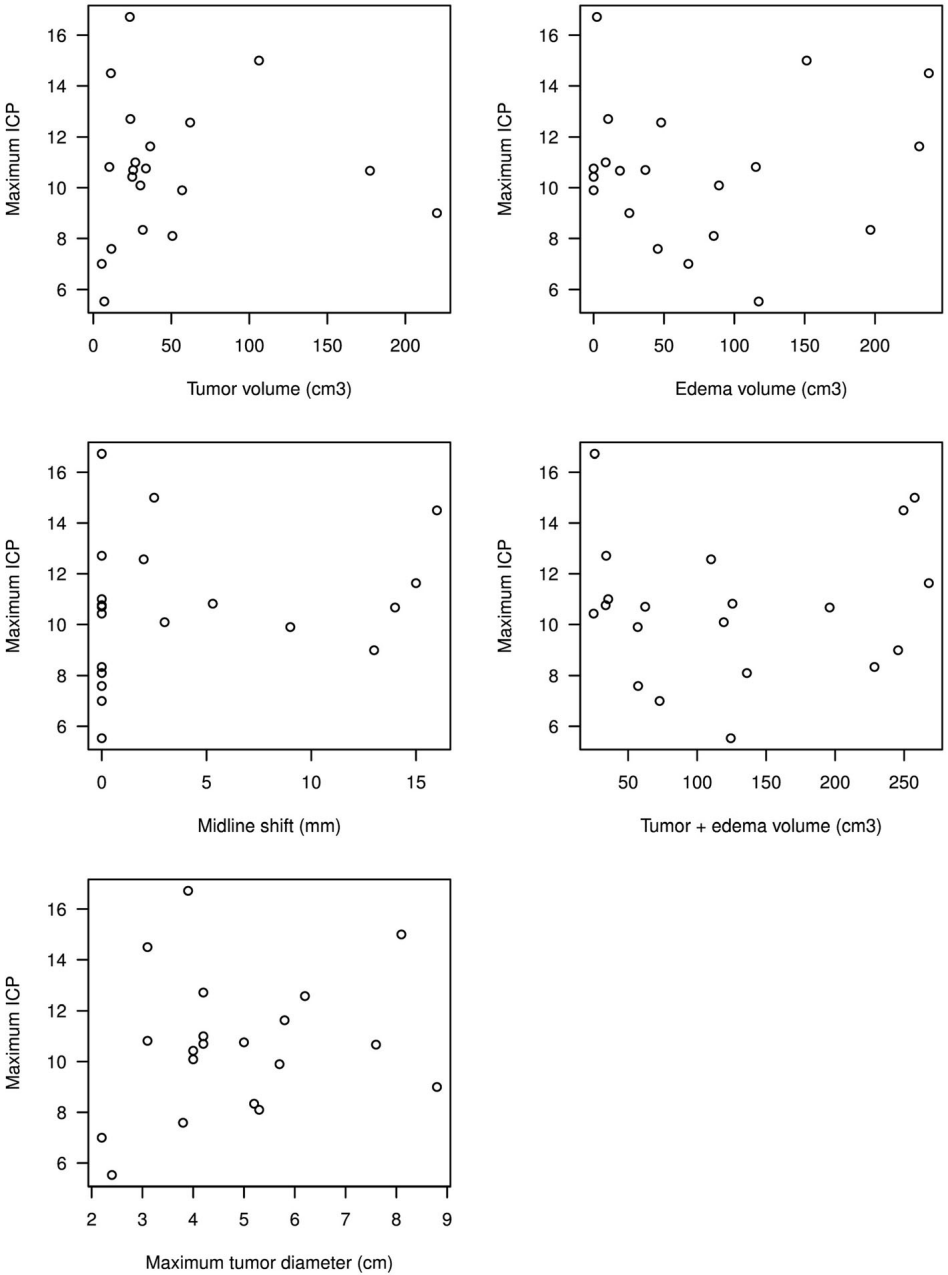
Scatterplots of maximum ICP vs. all continuous explanatory variables

The correlation of demographics, clinical and radiological variables in a bivariate association with the ICP_max_, ICP_mean_ and ICP_aff_, showed a statistically significant negative correlation between neurological deficits and both ICP_max_ (*p* = 0.02) and ICP_mean_ (*p* = 0.01), meaning that patients with neurological deficits had a − 5.81 mmHg lower ICP_max_ than patients without neurological deficits (Table 3, Fig. 3, Figs. 4 and 5). Noteworthy, that 90% of patients in our study cohort suffered from neurological deficits.

**Fig. 6:**
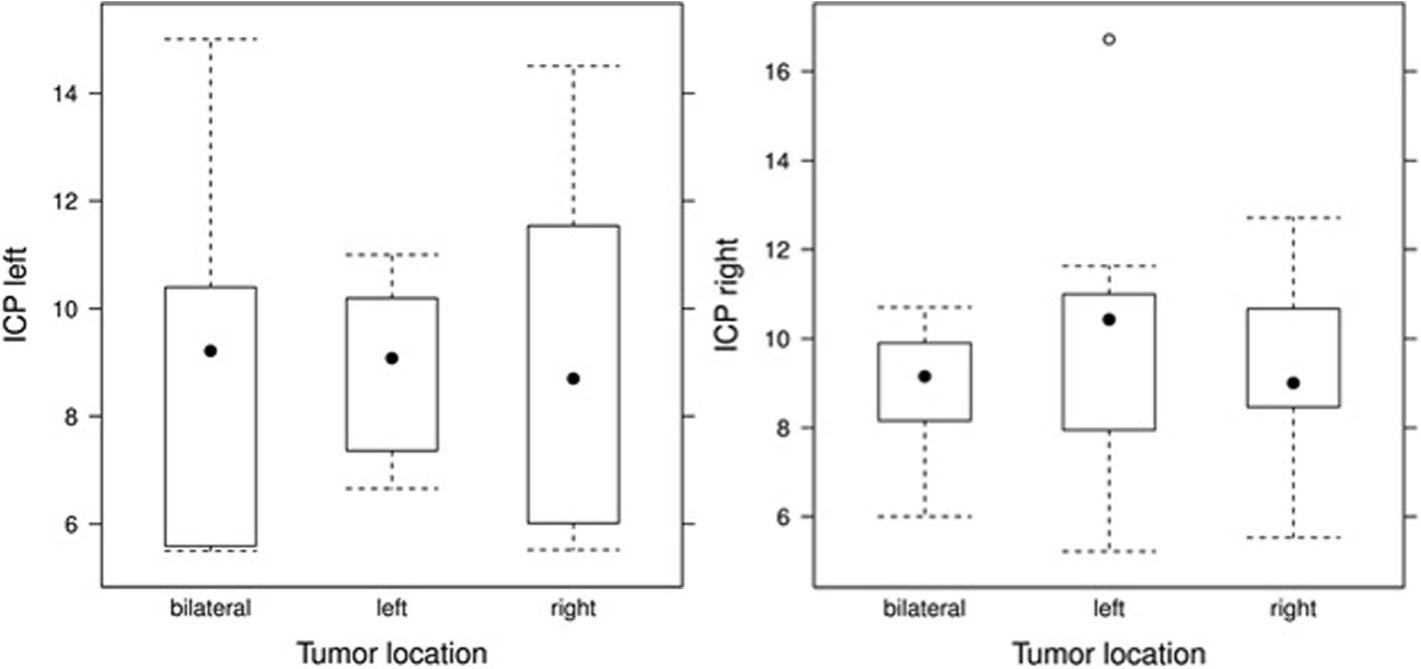
Boxplot of the ICP by tumor location **a**) ICP measured on left side **b**) ICP measured on right side

Neither the presence of a hydrocephalus, nor the intake of steroids showed any statistically significant correlation with ICP. Interestingly, ICP was not generally increased on the ipsilateral side of the tumor location (Fig. 6).

#### Multivariate model

The multivariate model selection for the primary endpoint ICP_max_ yielded neurological deficits and maximum tumor diameter as the only significant explanatory variables (Table 4). Neurological deficits were again associated with a lower ICP_max_. In contrast, maximum tumor diameter was associated with a higher ICP_max_. For instance, an increase of 1 cm in tumor diameter resulted in a 0.61 mmHg higher ICP. The same multivariate model was used for ICP_aff_ and ICP_mean_.

**Table 4:** Effect size estimates with 95% confidence intervals, t-values and *p*-values for the selected multivariate linear model for the ICP_max_, ICP_aff_, and ICP_mean_ per patient

	Variable	Effect size	95% CI	t-value	***p***-value
ICP_max_	Neurological deficits	−6.42	[−9.67, −3.17]	−4.16	**< 0.001**
	Max. tumor diameter	0.61	[0.05, 1.17]	2.31	**0.033**
ICP_aff_	Cerebrum vs. Cerebellum	−1.53	[−3.38, 0.32]	−1.75	0.099
	Max. tumor diameter (cm)	0.36	[−0.02, 0.75]	2.00	0.063
ICP_mean_	Neurological deficits	−5.93	[−8.12, −3.74]	−5.72	**< 0.001**
	Max. tumor diameter (cm)	0.36	[−0.01, 0.74]	2.04	0.057
ICP_all_	Neurological deficits	−4.84	[−7.63, −2.06]	−3.55	**0.0024**
	Steroids	−1.02	[−2.37, 0.33]	−1.55	0.1404

#### Analysis of all measurements

The results for the bivariate association analysis of explanatory variables with all ICP measurements is shown in (Table 4). Comparable with the analysis of ICP_max_ and ICP_mean_, the negative association of neurological deficits with ICP was the only statistically significant bivariate association.

## Discussion

### Relevance of this study

The relevance of this pilot study turns upon two observations. First, the availability of a non-invasive method of ICP measurement, which can be used in both the outpatient and the inpatient setting, allowing for a re-examination of a number of near-axiomatic beliefs about the dynamics of ICP in the face of various pathologic circumstances. Second, changes in our understanding of ICP dynamics may lead to advances in the nature and timing of therapy of space occupying lesions.

Surprisingly, the findings of our preliminary study contradict the widely-held belief that intracranial tumors necessarily induce intracranial hypertension as a consequence of mass effect and perifocal edema. Our observations, demonstrated only two statistically significant correlations: 1) a negative correlation with ICP and neurological deficits, and 2) a positive correlation between ICP and maximum tumor diameter. It was particularly interesting to note the negative correlation between ICP and neurological deficits. However, these results have to be interpreted carefully, because only 2 out of 20 patients had no neurological deficits. In a precise neurological examination almost all patients with mass effective brain tumor reveal some kind of neurological deficits. Therefore, this negative correlation might be influenced by observational factors such as sampling error. Additionally, it is also possible that patients with a neurological deficit self-selected and presented earlier, before increased intracranial pressure occurred.

So far, only a few case reports and series of brain tumor associated increased intracranial pressure have been reported. Tumor entities were metastasis [9], meningiomas [10–12], cavernous hemangioma [13], pineal [14] or dermoid cysts [15] infiltrating or compressing the superior sagittal sinus, transverse sinus or internal cerebral vein. This tumors lead to a sinus stenosis with rise in ICP, venous congestion and decreased CSF absorption [9–11, 13–15]. Intracranial hypertension was measured in 3 reports or defined by clinical signs in all other cases. Nevertheless, venous sinus thrombosis is known to cause an increase in ICP [16]. A simultaneous CSF and venous manometry showed, that a venous pressure rise can lead to a CSF absorption dysfunction, with increased CSF pressure [17].

Hung et al. [18] measured the ICP of 12 awake brain tumor patients in supine position prior to surgery with a mean ICP of 12.3 ± 6.4 mmHg, which is consistent with our results. Their study investigated the effect of 60° head rotation, which lead to an ICP increase up to 24.8 ± 14.3 mmHg, and elevation at 40° reduced ICP to − 0.2 ± 5.5 mmHg [18]. No clinical or radiological details on possible mass effect in these brain tumor patients were available.

### Are our standards for “normal ICP” correct?

Currently, we assume normal ICP value to be in a range between 7 and 15 mmHg, with the patient in 0° supine position [19]. In opposition to that, Andresen and Juhler published their results on ICP measurements with a telemetric ICP device in patients who underwent resection of a small brain tumor [20]. The ICP was measured 2 and 4 weeks postoperative when the ICP was supposed to be in normal range. Four weeks after surgery, mean ICP was 0.5 ± 4.0 mmHg in supine position and decreased to − 3.7 ± 3.8 mmHg in standing position [20]. These results suggest that ICP may be lower than previously estimated and that a negative ICP could be considered normal [20]. Intriguingly, if this hypothesis was proven to be true in larger studies, a mean ICP_max_ of 10.65 mmHg might qualify as increased, relatively speaking.

### The volume of space occupying lesions does not necessarily correlate with ICP

The Monro-Kellie doctrine describes certain aspects of pressure-volume-relationship: The volume-sum of brain, CSF and intracranial blood remains constant [21]. The latter act as buffers responding to volume increase by mass effective lesions, and are able to maintain a normal ICP and cerebral perfusion pressure (CPP) until the point of decompensation, when compliance is lost and ICP increases exponentially resulting in a CPP decrease [21].

The significance of our findings is that space-occupying lesions, surrounded by perifocal edema, do not necessarily result in increased intracranial pressure. A higher ICP was anticipated for intra-axial fast growing lesion, but this could not be confirmed. Meningioma grade I are slow growing extraaxial lesions, therefore giving the intracranial space more time for compensation mechanism of volume increase. On the contrary, high-grade gliomas are intraparenchymatous lesions and grow rapidly with a median growth rate of 0.14 cc per day and therefore shorter period for adjustment of regulatory mechanism [22].

### ICP ipsilateral and contralateral to space-occupying lesions

Furthermore, ipsi- and contralateral hemispheric ICP values were compared, assuming that we would expect a higher ICP on the side of the lesion. Contradictory, no correlation between ICP values and tumor lateralization could be found. Finally, tumor growth seems to affect both hemispheres equally.

### Steroid intake, edema and ICP

The mass effect in brain tumor cases is often not only caused by the tumor mass itself but mainly by peritumoral brain edema. For more than 5 decades, steroids are used in neurosurgical patients to reduce peritumoral brain edema (PTBE), for symptom relief, to reduce mass effect and risk of brain swelling during surgery [23].

For instance in meningiomas, approximately 60% of patients present with peritumoral edema [24]. In the study of Lobato et al., multivariate analysis revealed clinical predictors of cerebral edema including symptoms, seizures, and an intracranial hypertension syndrome [25, 26]. Other authors have demonstrated, that only the degree of cerebral edema surrounding the tumor correlates with ICP, but not tumor size or MLS [27]. In our study, no significant ICP correlation with edema, or intake of steroids could be demonstrated.

An earlier study investigated the ICP course after steroid treatment in patients with PTBE [1]. A total of 13 patients with brain tumors presenting with signs of increased ICP and a substantial amount of PTBE were included. ICP was measured with an epidural probe on the contralateral hemisphere during 5 days of methylprednisolone application [1]. Other than expected increased the initial mean ICP of 30.8 ± 22.9 mmHg significant to a mean ICP of 38.2 ± 19.8 mmHg in 7 (54%) patients [1]. Interestingly, steroid treatment led to a relevant rise of ICP in all patients with meningioma [1]. Other studies confirmed the poor response to corticosteroid therapy in meningioma [28–31]. Glioblastomas that respond superior to steroid therapy, have higher levels of corticosteroid receptors compared to meningiomas [32, 33]. Other authors suggested a decrease of edema and ICP after methylprednisolone treatment [34].

### Ventricular dilatation, hydrocephalus and ICP

Narotam et al. indicated in their study, that contralateral ventricular dilation is an early indicator of intracranial hypertension [35]. Indeed, there is no strong evidence, that chronic hydrocephalus causes ICP elevation. In different acute settings like aneurysmatic subarachnoid hemorrhage or colloid cyst associated hydrocephalus, ICP was found to be increased and CSF drainage was the immediate treatment for ICP reduction [36, 37]. The mechanism of acute deterioration seems to be associated with an increase in sagittal sinus pressure, which provokes acute brain swelling [37]. In chronic hydrocephalus patients, a modest chronic low-grade intracranial hypertension might be present [38]. In non-communicating, hydrocephalus, enlarged ventricles are thought to reflect increased ICP [39]. In fact, the ICP measurement in 2 patient groups with non-communicating hydrocephalus and 1) prior endoscopic third ventriculostomy or 2) no prior surgery, revealed a mean ICP of 9.7 mmHg, respectively 9.9 mmHg and no statistical significant association between ventricular volume and mean ICP could be proven. In accordance with these results, hydrocephalus was not associated with an increase in ICP in our study. Our patients developed an obstructive hydrocephalus due to the tumor growth, but were still compensating the increase in CSF volume.

### Limitations of the study

The main limitation of this pilot study is the low number of patients and the heterogeneity of histological diagnosis. Another aspect is that our measurements were only done at a certain time point and as ICP can be fluctuating, an increase in ICP might not be displayed. Continuous monitoring would therefore be necessary, which is not available with the non-invasive ICP measurement technique at this time. Although one patient in this series deteriorated before ICP measurement, our patients could finally still not have reached the point of decompensation and ICP increase.

Future studies are ongoing (ClinicalTrials.gov ID NCT03641443) to better characterize the dynamics of intracranial pressure in a larger series of patients presenting with brain tumor, and to assess applicability of non-invasive intracranial pressure monitoring.

## Conclusions

ICP in patients with intracranial tumors and mass effect is not proven to be increased. Clinical signs of intracranial hypertension do not necessarily reflect increased ICP, and therefore non-invasive ICP measurement might become an important tool in preoperative assessment of these patients in order to determine timing of surgery and the role of complementary medical interventions.

## Supplementary information

**Additional file 1.** Supplementary table with all obtained non-invasive ICP measurements in our cohort.

## Data Availability

All data generated or analyzed during this study are included in this published article and its supplementary information files.

## Abbreviations

CSF: Cerebrospinal Fluid
CPP: Cerebral Perfusion Pressure
GCS: Glasgow Comma Scale
ICP: Intracranial Pressure
MRI: Magnetic resonance imaging
MLS: Midline shift
OA: Ophthalmic artery
OC: Optic canal
PTBE: Peritumoral brain edema
TCD: Transcranial Doppler
