# Supplementary material for: Changing the paradigm of intracranial hypertension in brain tumor patients: a study based on non-invasive ICP measurements
Source: BMC Neurol. 2020 Jul 6;20:268. doi: 10.1186/s12883-020-01837-7 (PMC7336443; doi:10.1186/s12883-020-01837-7)
Supplement: Supplementary file 1 — Additional file 1. Supplementary table with all obtained non-invasive ICP measurements in our cohort. [file 12883_2020_1837_MOESM1_ESM.docx]

| Patient number | ICP left | ICP right |
| --- | --- | --- |
| 1 | 10.4 | 6 |
| 1 | 15 | N/A |
| 2 | 9.94 | 7.95 |
| 2 | 6.66 | 10.76 |
| 3 | 7.14 | 10.43 |
| 4 | 10.09 | 9.15 |
| 5 | 10.82 | N/A |
| 5 | 9.08 | N/A |
| 6 | 7 | N/A |
| 7 | 10.5 | 12.71 |
| 7 | 6.03 | 8.52 |
| 7 | 8 | 9.83 |
| 8 | 12.57 | 11.43 |
| 8 | 9.4 | 8.46 |
| 9 | 5.5 | 9.9 |
| 10 | 10.45 | 16.72 |
| 11 | 5.52 | 5.53 |
| 12 | 5.59 | 10.7 |
| 13 | 7.59 | 5.22 |
| 14 | 11 | 6 |
| 15 | 8 | 8.1 |
| 16 | 8.34 | 8.16 |
| 17 | N/A | 10.67 |
| 18 | 9.08 | 11 |
| 18 | N/A | 11.63 |
| 19 | 6 | 7.5 |
| 19 | N/A | 9 |
| 20 | 14.5 | N/A |
